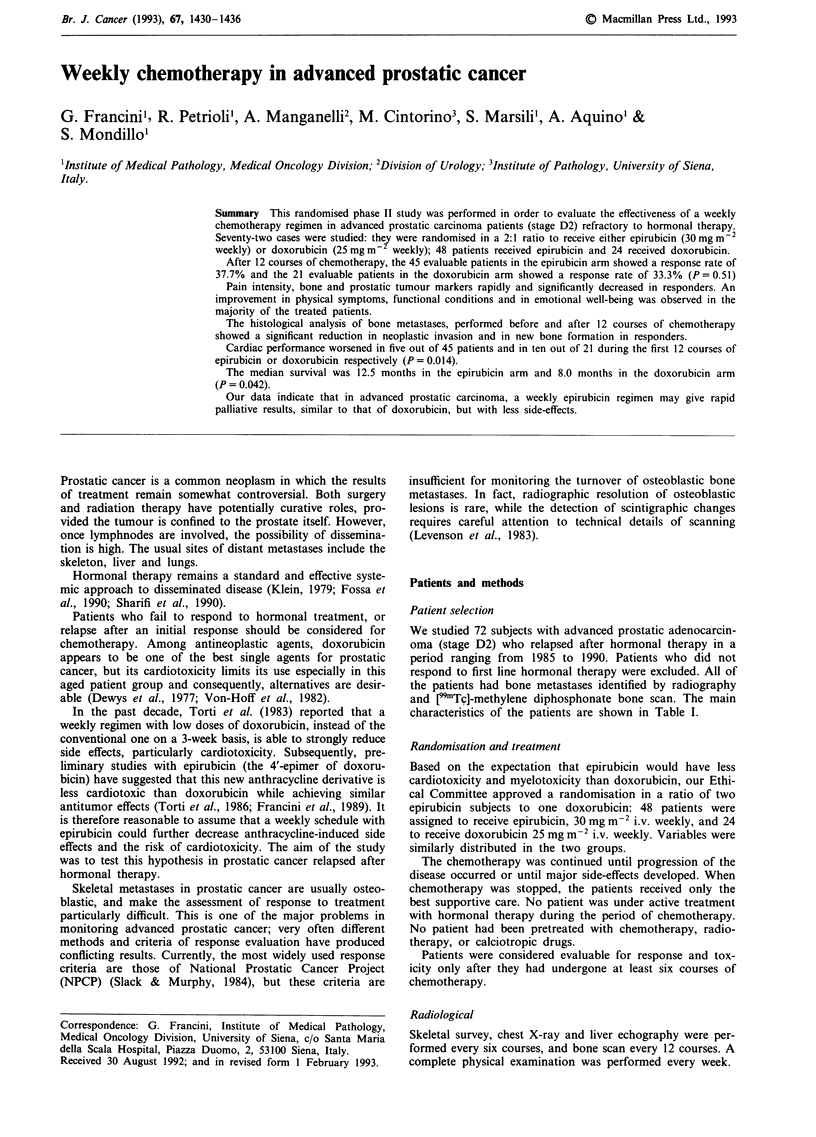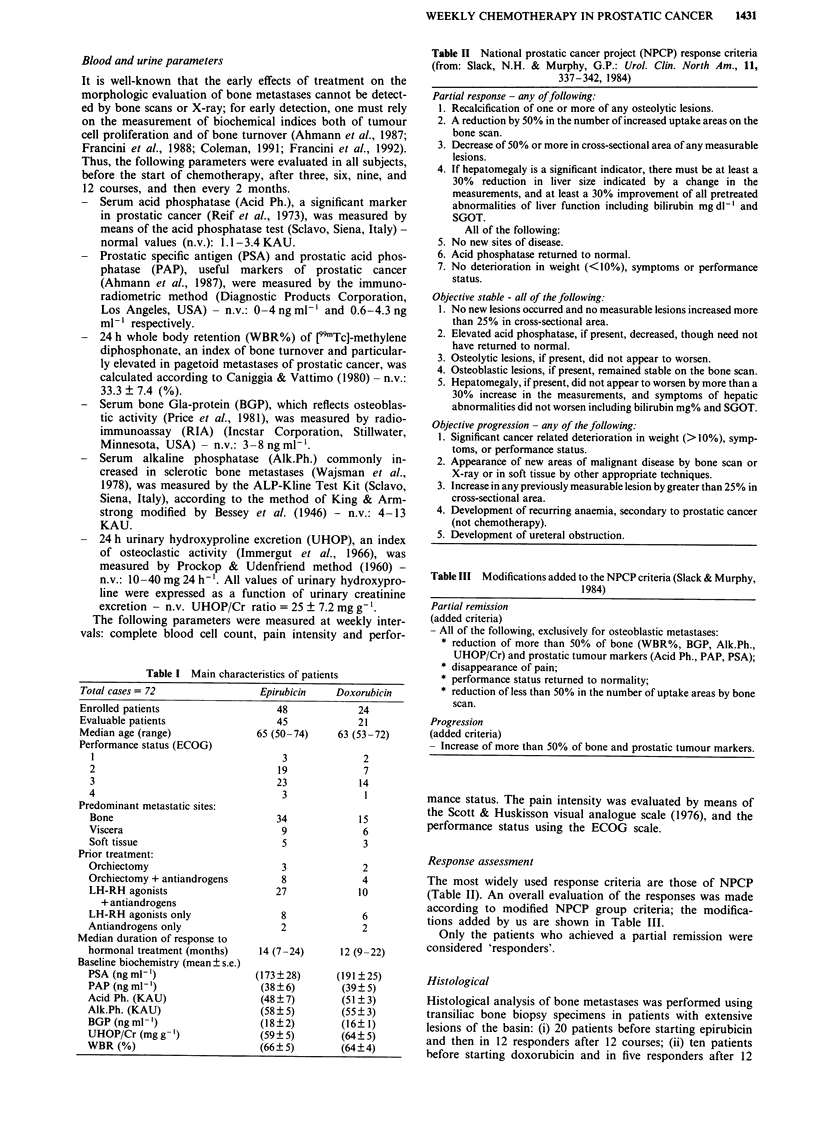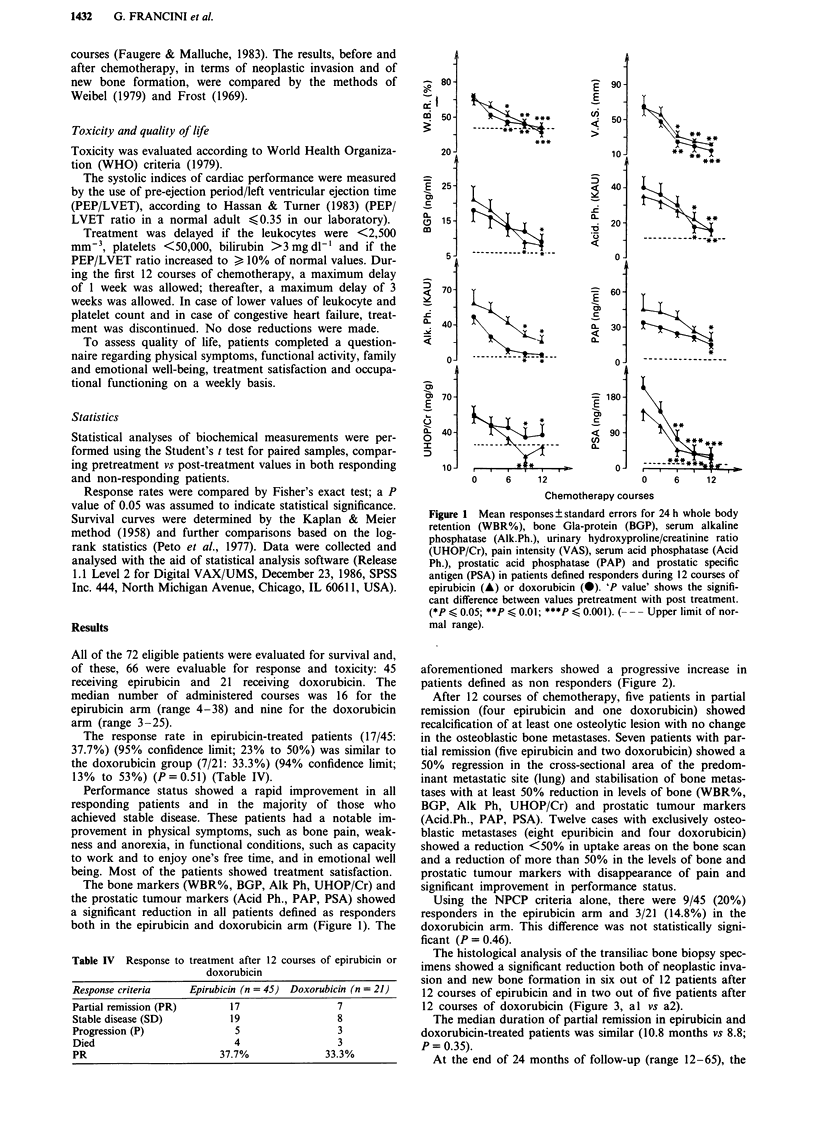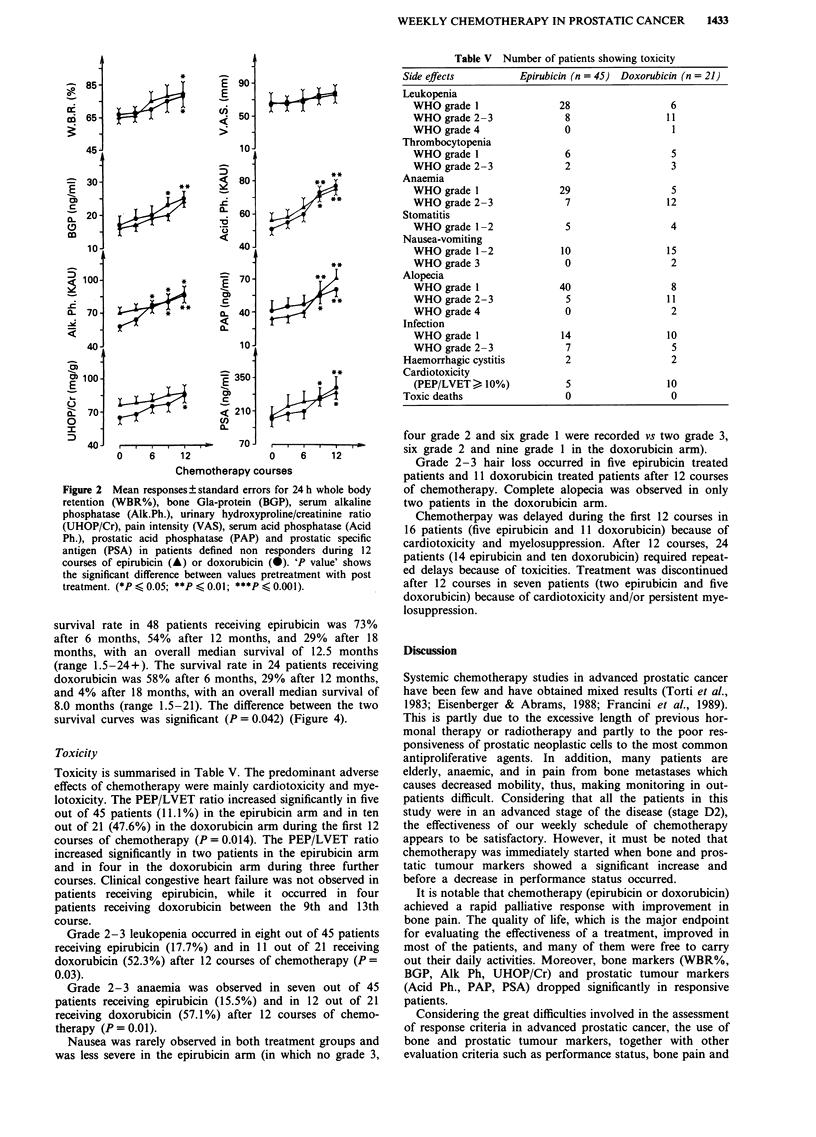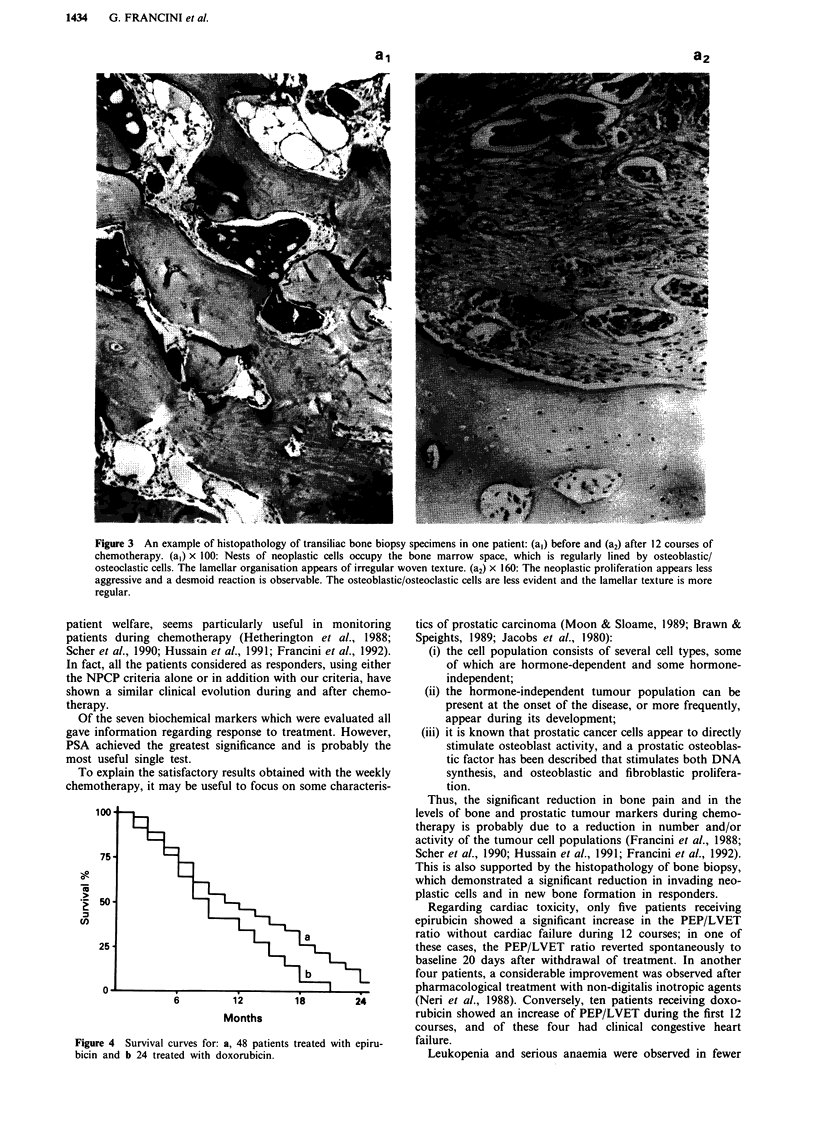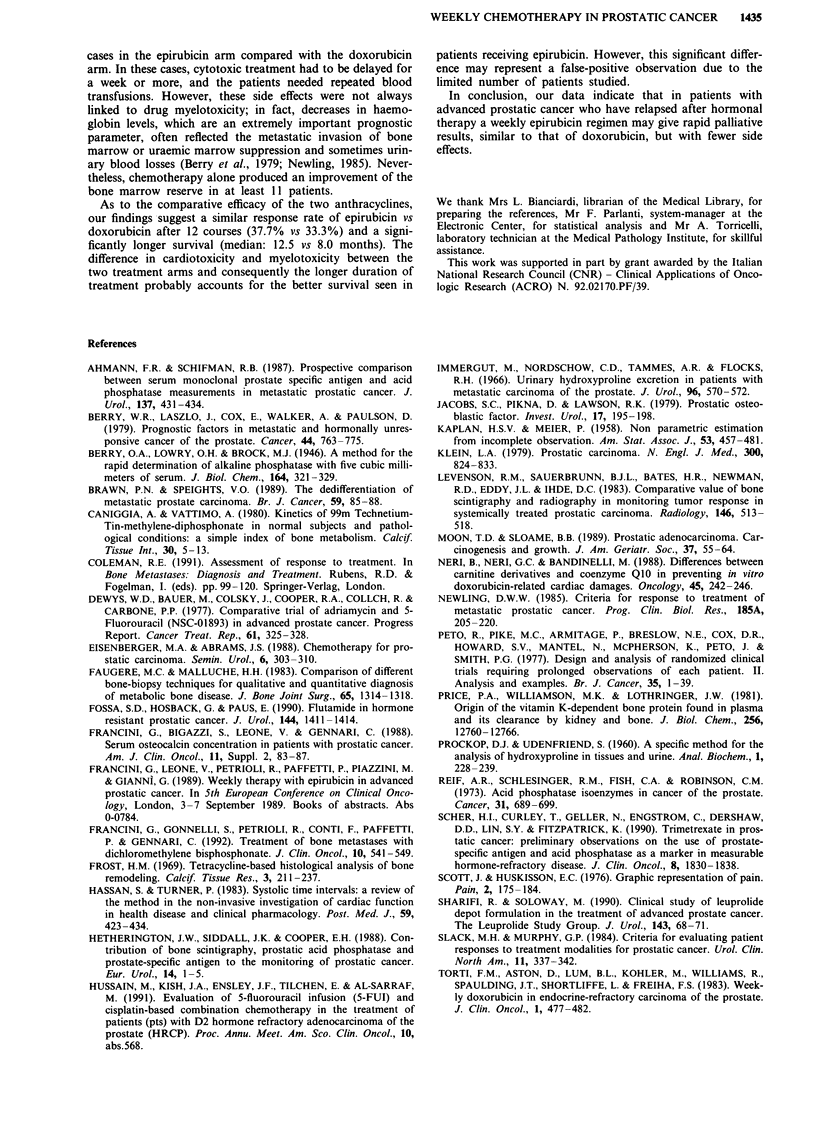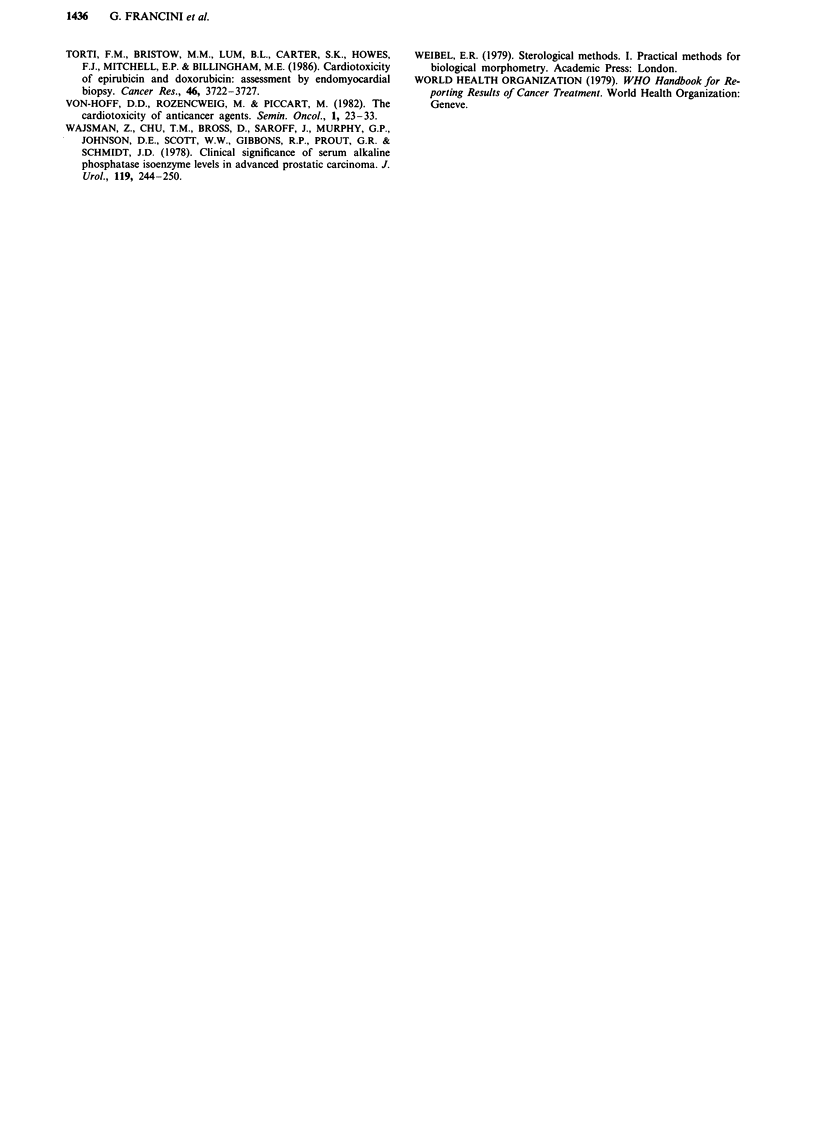# Weekly chemotherapy in advanced prostatic cancer.

**DOI:** 10.1038/bjc.1993.265

**Published:** 1993-06

**Authors:** G. Francini, R. Petrioli, A. Manganelli, M. Cintorino, S. Marsili, A. Aquino, S. Mondillo

**Affiliations:** Institute of Medical Pathology, University of Siena, Italy.

## Abstract

**Images:**


 © Macmillan Press Ltd., 1993

### Weekly chemotherapy in advanced prostatic cancer

G. Francini[1], R. Petrioli[1], A. Manganelli[2], M. Cintorino[3], S. Marsili[1], A. Aquino[1] & S. Mondillo[1]

[1]*Institute of Medical Pathology, Medical Oncology Division;* [2]*Division of Urology;* [3]*Institute of Pathology, University of Siena, Italy.*

**Summary** This randomised phase II study was performed in order to evaluate the effectiveness of a weekly chemotherapy regimen in advanced prostatic carcinoma patients (stage D2) refractory to hormonal therapy. Seventy-two cases were studied: they were randomised in a 2:1 ratio to receive either epirubicin (30 mg m$^{-2}$ weekly) or doxorubicin (25 mg m$^{-2}$ weekly); 48 patients received epirubicin and 24 received doxorubicin.

After 12 courses of chemotherapy, the 45 evaluable patients in the epirubicin arm showed a response rate of 37.7% and the 21 evaluable patients in the doxorubicin arm showed a response rate of 33.3% ($P = 0.51$)

Pain intensity, bone and prostatic tumour markers rapidly and significantly decreased in responders. An improvement in physical symptoms, functional conditions and in emotional well-being was observed in the majority of the treated patients.

The histological analysis of bone metastases, performed before and after 12 courses of chemotherapy showed a significant reduction in neoplastic invasion and in new bone formation in responders.

Cardiac performance worsened in five out of 45 patients and in ten out of 21 during the first 12 courses of epirubicin or doxorubicin respectively ($P = 0.014$).

The median survival was 12.5 months in the epirubicin arm and 8.0 months in the doxorubicin arm ($P = 0.042$).

Our data indicate that in advanced prostatic carcinoma, a weekly epirubicin regimen may give rapid palliative results, similar to that of doxorubicin, but with less side-effects.

Prostatic cancer is a common neoplasm in which the results of treatment remain somewhat controversial. Both surgery and radiation therapy have potentially curative roles, provided the tumour is confined to the prostate itself. However, once lymphnodes are involved, the possibility of dissemination is high. The usual sites of distant metastases include the skeleton, liver and lungs.

Hormonal therapy remains a standard and effective systemic approach to disseminated disease (Klein, 1979; Fossa *et al.*, 1990; Sharifi *et al.*, 1990).

Patients who fail to respond to hormonal treatment, or relapse after an initial response should be considered for chemotherapy. Among antineoplastic agents, doxorubicin appears to be one of the best single agents for prostatic cancer, but its cardiotoxicity limits its use especially in this aged patient group and consequently, alternatives are desirable (Dewys *et al.*, 1977; Von-Hoff *et al.*, 1982).

In the past decade, Torti *et al.* (1983) reported that a weekly regimen with low doses of doxorubicin, instead of the conventional one on a 3-week basis, is able to strongly reduce side effects, particularly cardiotoxicity. Subsequently, preliminary studies with epirubicin (the 4′-epimer of doxorubicin) have suggested that this new anthracycline derivative is less cardiotoxic than doxorubicin while achieving similar antitumor effects (Torti *et al.*, 1986; Francini *et al.*, 1989). It is therefore reasonable to assume that a weekly schedule with epirubicin could further decrease anthracycline-induced side effects and the risk of cardiotoxicity. The aim of the study was to test this hypothesis in prostatic cancer relapsed after hormonal therapy.

Skeletal metastases in prostatic cancer are usually osteoblastic, and make the assessment of response to treatment particularly difficult. This is one of the major problems in monitoring advanced prostatic cancer; very often different methods and criteria of response evaluation have produced conflicting results. Currently, the most widely used response criteria are those of National Prostatic Cancer Project (NPCP) (Slack & Murphy, 1984), but these criteria are insufficient for monitoring the turnover of osteoblastic bone metastases. In fact, radiographic resolution of osteoblastic lesions is rare, while the detection of scintigraphic changes requires careful attention to technical details of scanning (Levenson *et al.*, 1983).

### Patients and methods

### Patient selection

We studied 72 subjects with advanced prostatic adenocarcinoma (stage D2) who relapsed after hormonal therapy in a period ranging from 1985 to 1990. Patients who did not respond to first line hormonal therapy were excluded. All of the patients had bone metastases identified by radiography and [$^{99m}$Tc]-methylene diphosphonate bone scan. The main characteristics of the patients are shown in Table I.

### Randomisation and treatment

Based on the expectation that epirubicin would have less cardiotoxicity and myelotoxicity than doxorubicin, our Ethical Committee approved a randomisation in a ratio of two epirubicin subjects to one doxorubicin: 48 patients were assigned to receive epirubicin, 30 mg m$^{-2}$ i.v. weekly, and 24 to receive doxorubicin 25 mg m$^{-2}$ i.v. weekly. Variables were similarly distributed in the two groups.

The chemotherapy was continued until progression of the disease occurred or until major side-effects developed. When chemotherapy was stopped, the patients received only the best supportive care. No patient was under active treatment with hormonal therapy during the period of chemotherapy. No patient had been pretreated with chemotherapy, radiotherapy, or calciotropic drugs.

Patients were considered evaluable for response and toxicity only after they had undergone at least six courses of chemotherapy.

### Radiological

Skeletal survey, chest X-ray and liver echography were performed every six courses, and bone scan every 12 courses. A complete physical examination was performed every week.

Correspondence: G. Francini, Institute of Medical Pathology, Medical Oncology Division, University of Siena, c/o Santa Maria della Scala Hospital, Piazza Duomo, 2, 53100 Siena, Italy.
Received 30 August 1992; and in revised form 1 February 1993.

*Blood and urine parameters*

It is well-known that the early effects of treatment on the morphologic evaluation of bone metastases cannot be detected by bone scans or X-ray; for early detection, one must rely on the measurement of biochemical indices both of tumour cell proliferation and of bone turnover (Ahmann *et al.*, 1987; Francini *et al.*, 1988; Coleman, 1991; Francini *et al.*, 1992). Thus, the following parameters were evaluated in all subjects, before the start of chemotherapy, after three, six, nine, and 12 courses, and then every 2 months.

- Serum acid phosphatase (Acid Ph.), a significant marker in prostatic cancer (Reif *et al.*, 1973), was measured by means of the acid phosphatase test (Sclavo, Siena, Italy) – normal values (n.v.): 1.1–3.4 KAU.
- Prostatic specific antigen (PSA) and prostatic acid phosphatase (PAP), useful markers of prostatic cancer (Ahmann *et al.*, 1987), were measured by the immunoradiometric method (Diagnostic Products Corporation, Los Angeles, USA) – n.v.: 0–4 ng $ml^{-1}$ and 0.6–4.3 ng $ml^{-1}$ respectively.
- 24 h whole body retention (WBR%) of [$^{99m}$Tc]-methylene diphosphonate, an index of bone turnover and particularly elevated in pagetoid metastases of prostatic cancer, was calculated according to Caniggia & Vattimo (1980) – n.v.: 33.3 ± 7.4 (%).
- Serum bone Gla-protein (BGP), which reflects osteoblastic activity (Price *et al.*, 1981), was measured by radioimmunoassay (RIA) (Incstar Corporation, Stillwater, Minnesota, USA) – n.v.: 3–8 ng $ml^{-1}$.
- Serum alkaline phosphatase (Alk.Ph.) commonly increased in sclerotic bone metastases (Wajsman *et al.*, 1978), was measured by the ALP-Kline Test Kit (Sclavo, Siena, Italy), according to the method of King & Armstrong modified by Bessey *et al.* (1946) – n.v.: 4–13 KAU.
- 24 h urinary hydroxyproline excretion (UHOP), an index of osteoclastic activity (Immergut *et al.*, 1966), was measured by Prockop & Udenfriend method (1960) – n.v.: 10–40 mg 24 $h^{-1}$. All values of urinary hydroxyproline were expressed as a function of urinary creatinine excretion – n.v. UHOP/Cr ratio = 25 ± 7.2 mg $g^{-1}$.

The following parameters were measured at weekly intervals: complete blood cell count, pain intensity and performance status. The pain intensity was evaluated by means of the Scott & Huskisson visual analogue scale (1976), and the performance status using the ECOG scale.

**Table I** Main characteristics of patients

| *Total cases = 72* | *Epirubicin* | *Doxorubicin* |
|---|---|---|
| Enrolled patients | 48 | 24 |
| Evaluable patients | 45 | 21 |
| Median age (range) | 65 (50–74) | 63 (53–72) |
| Performance status (ECOG) | | |
| 1 | 3 | 2 |
| 2 | 19 | 7 |
| 3 | 23 | 14 |
| 4 | 3 | 1 |
| Predominant metastatic sites: | | |
| Bone | 34 | 15 |
| Viscera | 9 | 6 |
| Soft tissue | 5 | 3 |
| Prior treatment: | | |
| Orchiectomy | 3 | 2 |
| Orchiectomy + antiandrogens | 8 | 4 |
| LH-RH agonists + antiandrogens | 27 | 10 |
| LH-RH agonists only | 8 | 6 |
| Antiandrogens only | 2 | 2 |
| Median duration of response to hormonal treatment (months) | 14 (7–24) | 12 (9–22) |
| Baseline biochemistry (mean ± s.e.) | | |
| PSA (ng $ml^{-1}$) | (173 ± 28) | (191 ± 25) |
| PAP (ng $ml^{-1}$) | (38 ± 6) | (39 ± 5) |
| Acid Ph. (KAU) | (48 ± 7) | (51 ± 3) |
| Alk.Ph. (KAU) | (58 ± 5) | (55 ± 3) |
| BGP (ng $ml^{-1}$) | (18 ± 2) | (16 ± 1) |
| UHOP/Cr (mg $g^{-1}$) | (59 ± 5) | (64 ± 5) |
| WBR (%) | (66 ± 5) | (64 ± 4) |

**Table II** National prostatic cancer project (NPCP) response criteria (from: Slack, N.H. & Murphy, G.P.: *Urol. Clin. North Am.*, **11**, 337–342, 1984)

*Partial response – any of following:*
1. Recalcification of one or more of any osteolytic lesions.
2. A reduction by 50% in the number of increased uptake areas on the bone scan.
3. Decrease of 50% or more in cross-sectional area of any measurable lesions.
4. If hepatomegaly is a significant indicator, there must be at least a 30% reduction in liver size indicated by a change in the measurements, and at least a 30% improvement of all pretreated abnormalities of liver function including bilirubin mg $dl^{-1}$ and SGOT.

All of the following:

5. No new sites of disease.
6. Acid phosphatase returned to normal.
7. No deterioration in weight (<10%), symptoms or performance status.

*Objective stable - all of the following:*
1. No new lesions occurred and no measurable lesions increased more than 25% in cross-sectional area.
2. Elevated acid phosphatase, if present, decreased, though need not have returned to normal.
3. Osteolytic lesions, if present, did not appear to worsen.
4. Osteoblastic lesions, if present, remained stable on the bone scan.
5. Hepatomegaly, if present, did not appear to worsen by more than a 30% increase in the measurements, and symptoms of hepatic abnormalities did not worsen including bilirubin mg% and SGOT.

*Objective progression – any of the following:*
1. Significant cancer related deterioration in weight (>10%), symptoms, or performance status.
2. Appearance of new areas of malignant disease by bone scan or X-ray or in soft tissue by other appropriate techniques.
3. Increase in any previously measurable lesion by greater than 25% in cross-sectional area.
4. Development of recurring anaemia, secondary to prostatic cancer (not chemotherapy).
5. Development of ureteral obstruction.

**Table III** Modifications added to the NPCP criteria (Slack & Murphy, 1984)

*Partial remission*
(added criteria)
– All of the following, exclusively for osteoblastic metastases:
* reduction of more than 50% of bone (WBR%, BGP, Alk.Ph., UHOP/Cr) and prostatic tumour markers (Acid Ph., PAP, PSA);
* disappearance of pain;
* performance status returned to normality;
* reduction of less than 50% in the number of uptake areas by bone scan.

*Progression*
(added criteria)
– Increase of more than 50% of bone and prostatic tumour markers.

*Response assessment*

The most widely used response criteria are those of NPCP (Table II). An overall evaluation of the responses was made according to modified NPCP group criteria; the modifications added by us are shown in Table III.

Only the patients who achieved a partial remission were considered 'responders'.

*Histological*

Histological analysis of bone metastases was performed using transiliac bone biopsy specimens in patients with extensive lesions of the basin: (i) 20 patients before starting epirubicin and then in 12 responders after 12 courses; (ii) ten patients before starting doxorubicin and in five responders after 12

courses (Faugere & Malluche, 1983). The results, before and after chemotherapy, in terms of neoplastic invasion and of new bone formation, were compared by the methods of Weibel (1979) and Frost (1969).

*Toxicity and quality of life*

Toxicity was evaluated according to World Health Organization (WHO) criteria (1979).

The systolic indices of cardiac performance were measured by the use of pre-ejection period/left ventricular ejection time (PEP/LVET), according to Hassan & Turner (1983) (PEP/LVET ratio in a normal adult $\leqslant 0.35$ in our laboratory).

Treatment was delayed if the leukocytes were $<2,500$ $mm^{-3}$, platelets $<50,000$, bilirubin $>3\ mg\ dl^{-1}$ and if the PEP/LVET ratio increased to $\geqslant 10\%$ of normal values. During the first 12 courses of chemotherapy, a maximum delay of 1 week was allowed; thereafter, a maximum delay of 3 weeks was allowed. In case of lower values of leukocyte and platelet count and in case of congestive heart failure, treatment was discontinued. No dose reductions were made.

To assess quality of life, patients completed a questionnaire regarding physical symptoms, functional activity, family and emotional well-being, treatment satisfaction and occupational functioning on a weekly basis.

*Statistics*

Statistical analyses of biochemical measurements were performed using the Student's *t* test for paired samples, comparing pretreatment *vs* post-treatment values in both responding and non-responding patients.

Response rates were compared by Fisher's exact test; a *P* value of 0.05 was assumed to indicate statistical significance. Survival curves were determined by the Kaplan & Meier method (1958) and further comparisons based on the log-rank statistics (Peto *et al.*, 1977). Data were collected and analysed with the aid of statistical analysis software (Release 1.1 Level 2 for Digital VAX/UMS, December 23, 1986, SPSS Inc. 444, North Michigan Avenue, Chicago, IL 60611, USA).

### Results

All of the 72 eligible patients were evaluated for survival and, of these, 66 were evaluable for response and toxicity: 45 receiving epirubicin and 21 receiving doxorubicin. The median number of administered courses was 16 for the epirubicin arm (range 4–38) and nine for the doxorubicin arm (range 3–25).

The response rate in epirubicin-treated patients (17/45: 37.7%) (95% confidence limit; 23% to 50%) was similar to the doxorubicin group (7/21: 33.3%) (94% confidence limit; 13% to 53%) ($P = 0.51$) (Table IV).

Performance status showed a rapid improvement in all responding patients and in the majority of those who achieved stable disease. These patients had a notable improvement in physical symptoms, such as bone pain, weakness and anorexia, in functional conditions, such as capacity to work and to enjoy one's free time, and in emotional well being. Most of the patients showed treatment satisfaction.

The bone markers (WBR%, BGP, Alk Ph, UHOP/Cr) and the prostatic tumour markers (Acid Ph., PAP, PSA) showed a significant reduction in all patients defined as responders both in the epirubicin and doxorubicin arm (Figure 1). The

**Table IV** Response to treatment after 12 courses of epirubicin or doxorubicin

| *Response criteria* | *Epirubicin (n = 45)* | *Doxorubicin (n = 21)* |
|---|---|---|
| Partial remission (PR) | 17 | 7 |
| Stable disease (SD) | 19 | 8 |
| Progression (P) | 5 | 3 |
| Died | 4 | 3 |
| PR | 37.7% | 33.3% |

**Figure 1** Mean responses ± standard errors for 24 h whole body retention (WBR%), bone Gla-protein (BGP), serum alkaline phosphatase (Alk.Ph.), urinary hydroxyproline/creatinine ratio (UHOP/Cr), pain intensity (VAS), serum acid phosphatase (Acid Ph.), prostatic acid phosphatase (PAP) and prostatic specific antigen (PSA) in patients defined responders during 12 courses of epirubicin (▲) or doxorubicin (●). '*P* value' shows the significant difference between values pretreatment with post treatment. (**P* ⩽ 0.05; ***P* ⩽ 0.01; ****P* ⩽ 0.001). (--- Upper limit of normal range).

aforementioned markers showed a progressive increase in patients defined as non responders (Figure 2).

After 12 courses of chemotherapy, five patients in partial remission (four epirubicin and one doxorubicin) showed recalcification of at least one osteolytic lesion with no change in the osteoblastic bone metastases. Seven patients with partial remission (five epirubicin and two doxorubicin) showed a 50% regression in the cross-sectional area of the predominant metastatic site (lung) and stabilisation of bone metastases with at least 50% reduction in levels of bone (WBR%, BGP, Alk Ph, UHOP/Cr) and prostatic tumour markers (Acid.Ph., PAP, PSA). Twelve cases with exclusively osteoblastic metastases (eight epuribicin and four doxorubicin) showed a reduction <50% in uptake areas on the bone scan and a reduction of more than 50% in the levels of bone and prostatic tumour markers with disappearance of pain and significant improvement in performance status.

Using the NPCP criteria alone, there were 9/45 (20%) responders in the epirubicin arm and 3/21 (14.8%) in the doxorubicin arm. This difference was not statistically significant ($P = 0.46$).

The histological analysis of the transiliac bone biopsy specimens showed a significant reduction both of neoplastic invasion and new bone formation in six out of 12 patients after 12 courses of epirubicin and in two out of five patients after 12 courses of doxorubicin (Figure 3, a1 *vs* a2).

The median duration of partial remission in epirubicin and doxorubicin-treated patients was similar (10.8 months *vs* 8.8; $P = 0.35$).

At the end of 24 months of follow-up (range 12–65), the

**Figure 2** Mean responses ± standard errors for 24 h whole body retention (WBR%), bone Gla-protein (BGP), serum alkaline phosphatase (Alk.Ph.), urinary hydroxyproline/creatinine ratio (UHOP/Cr), pain intensity (VAS), serum acid phosphatase (Acid Ph.), prostatic acid phosphatase (PAP) and prostatic specific antigen (PSA) in patients defined non responders during 12 courses of epirubicin (▲) or doxorubicin (●). '*P* value' shows the significant difference between values pretreatment with post treatment. (*$P \leq 0.05$; **$P \leq 0.01$; ***$P \leq 0.001$).

survival rate in 48 patients receiving epirubicin was 73% after 6 months, 54% after 12 months, and 29% after 18 months, with an overall median survival of 12.5 months (range 1.5–24+). The survival rate in 24 patients receiving doxorubicin was 58% after 6 months, 29% after 12 months, and 4% after 18 months, with an overall median survival of 8.0 months (range 1.5–21). The difference between the two survival curves was significant ($P = 0.042$) (Figure 4).

### *Toxicity*

Toxicity is summarised in Table V. The predominant adverse effects of chemotherapy were mainly cardiotoxicity and myelotoxicity. The PEP/LVET ratio increased significantly in five out of 45 patients (11.1%) in the epirubicin arm and in ten out of 21 (47.6%) in the doxorubicin arm during the first 12 courses of chemotherapy ($P = 0.014$). The PEP/LVET ratio increased significantly in two patients in the epirubicin arm and in four in the doxorubicin arm during three further courses. Clinical congestive heart failure was not observed in patients receiving epirubicin, while it occurred in four patients receiving doxorubicin between the 9th and 13th course.

Grade 2–3 leukopenia occurred in eight out of 45 patients receiving epirubicin (17.7%) and in 11 out of 21 receiving doxorubicin (52.3%) after 12 courses of chemotherapy ($P = 0.03$).

Grade 2–3 anaemia was observed in seven out of 45 patients receiving epirubicin (15.5%) and in 12 out of 21 receiving doxorubicin (57.1%) after 12 courses of chemotherapy ($P = 0.01$).

Nausea was rarely observed in both treatment groups and was less severe in the epirubicin arm (in which no grade 3, four grade 2 and six grade 1 were recorded *vs* two grade 3, six grade 2 and nine grade 1 in the doxorubicin arm).

Grade 2–3 hair loss occurred in five epirubicin treated patients and 11 doxorubicin treated patients after 12 courses of chemotherapy. Complete alopecia was observed in only two patients in the doxorubicin arm.

Chemotherpay was delayed during the first 12 courses in 16 patients (five epirubicin and 11 doxorubicin) because of cardiotoxicity and myelosuppression. After 12 courses, 24 patients (14 epirubicin and ten doxorubicin) required repeated delays because of toxicities. Treatment was discontinued after 12 courses in seven patients (two epirubicin and five doxorubicin) because of cardiotoxicity and/or persistent myelosuppression.

**Table V** Number of patients showing toxicity

| *Side effects* | *Epirubicin (n = 45)* | *Doxorubicin (n = 21)* |
|---|---|---|
| Leukopenia | | |
| WHO grade 1 | 28 | 6 |
| WHO grade 2–3 | 8 | 11 |
| WHO grade 4 | 0 | 1 |
| Thrombocytopenia | | |
| WHO grade 1 | 6 | 5 |
| WHO grade 2–3 | 2 | 3 |
| Anaemia | | |
| WHO grade 1 | 29 | 5 |
| WHO grade 2–3 | 7 | 12 |
| Stomatitis | | |
| WHO grade 1–2 | 5 | 4 |
| Nausea-vomiting | | |
| WHO grade 1–2 | 10 | 15 |
| WHO grade 3 | 0 | 2 |
| Alopecia | | |
| WHO grade 1 | 40 | 8 |
| WHO grade 2–3 | 5 | 11 |
| WHO grade 4 | 0 | 2 |
| Infection | | |
| WHO grade 1 | 14 | 10 |
| WHO grade 2–3 | 7 | 5 |
| Haemorrhagic cystitis | 2 | 2 |
| Cardiotoxicity | | |
| (PEP/LVET ≥ 10%) | 5 | 10 |
| Toxic deaths | 0 | 0 |

### Discussion

Systemic chemotherapy studies in advanced prostatic cancer have been few and have obtained mixed results (Torti *et al.*, 1983; Eisenberger & Abrams, 1988; Francini *et al.*, 1989). This is partly due to the excessive length of previous hormonal therapy or radiotherapy and partly to the poor responsiveness of prostatic neoplastic cells to the most common antiproliferative agents. In addition, many patients are elderly, anaemic, and in pain from bone metastases which causes decreased mobility, thus, making monitoring in outpatients difficult. Considering that all the patients in this study were in an advanced stage of the disease (stage D2), the effectiveness of our weekly schedule of chemotherapy appears to be satisfactory. However, it must be noted that chemotherapy was immediately started when bone and prostatic tumour markers showed a significant increase and before a decrease in performance status occurred.

It is notable that chemotherapy (epirubicin or doxorubicin) achieved a rapid palliative response with improvement in bone pain. The quality of life, which is the major endpoint for evaluating the effectiveness of a treatment, improved in most of the patients, and many of them were free to carry out their daily activities. Moreover, bone markers (WBR%, BGP, Alk Ph, UHOP/Cr) and prostatic tumour markers (Acid Ph., PAP, PSA) dropped significantly in responsive patients.

Considering the great difficulties involved in the assessment of response criteria in advanced prostatic cancer, the use of bone and prostatic tumour markers, together with other evaluation criteria such as performance status, bone pain and

**Figure 3** An example of histopathology of transiliac bone biopsy specimens in one patient: ($a_1$) before and ($a_2$) after 12 courses of chemotherapy. ($a_1$) × 100: Nests of neoplastic cells occupy the bone marrow space, which is regularly lined by osteoblastic/osteoclastic cells. The lamellar organisation appears of irregular woven texture. ($a_2$) × 160: The neoplastic proliferation appears less aggressive and a desmoid reaction is observable. The osteoblastic/osteoclastic cells are less evident and the lamellar texture is more regular.

patient welfare, seems particularly useful in monitoring patients during chemotherapy (Hetherington *et al.*, 1988; Scher *et al.*, 1990; Hussain *et al.*, 1991; Francini *et al.*, 1992). In fact, all the patients considered as responders, using either the NPCP criteria alone or in addition with our criteria, have shown a similar clinical evolution during and after chemotherapy.

Of the seven biochemical markers which were evaluated all gave information regarding response to treatment. However, PSA achieved the greatest significance and is probably the most useful single test.

To explain the satisfactory results obtained with the weekly chemotherapy, it may be useful to focus on some characteristics of prostatic carcinoma (Moon & Sloame, 1989; Brawn & Speights, 1989; Jacobs *et al.*, 1980):

(i) the cell population consists of several cell types, some of which are hormone-dependent and some hormone-independent;
(ii) the hormone-independent tumour population can be present at the onset of the disease, or more frequently, appear during its development;
(iii) it is known that prostatic cancer cells appear to directly stimulate osteoblast activity, and a prostatic osteoblastic factor has been described that stimulates both DNA synthesis, and osteoblastic and fibroblastic proliferation.

Thus, the significant reduction in bone pain and in the levels of bone and prostatic tumour markers during chemotherapy is probably due to a reduction in number and/or activity of the tumour cell populations (Francini *et al.*, 1988; Scher *et al.*, 1990; Hussain *et al.*, 1991; Francini *et al.*, 1992). This is also supported by the histopathology of bone biopsy, which demonstrated a significant reduction in invading neoplastic cells and in new bone formation in responders.

Regarding cardiac toxicity, only five patients receiving epirubicin showed a significant increase in the PEP/LVET ratio without cardiac failure during 12 courses; in one of these cases, the PEP/LVET ratio reverted spontaneously to baseline 20 days after withdrawal of treatment. In another four patients, a considerable improvement was observed after pharmacological treatment with non-digitalis inotropic agents (Neri *et al.*, 1988). Conversely, ten patients receiving doxorubicin showed an increase of PEP/LVET during the first 12 courses, and of these four had clinical congestive heart failure.

Leukopenia and serious anaemia were observed in fewer

**Figure 4** Survival curves for: **a**, 48 patients treated with epirubicin and **b** 24 treated with doxorubicin.

cases in the epirubicin arm compared with the doxorubicin arm. In these cases, cytotoxic treatment had to be delayed for a week or more, and the patients needed repeated blood transfusions. However, these side effects were not always linked to drug myelotoxicity; in fact, decreases in haemoglobin levels, which are an extremely important prognostic parameter, often reflected the metastatic invasion of bone marrow or uraemic marrow suppression and sometimes urinary blood losses (Berry *et al.*, 1979; Newling, 1985). Nevertheless, chemotherapy alone produced an improvement of the bone marrow reserve in at least 11 patients.

As to the comparative efficacy of the two anthracyclines, our findings suggest a similar response rate of epirubicin *vs* doxorubicin after 12 courses (37.7% *vs* 33.3%) and a significantly longer survival (median: 12.5 *vs* 8.0 months). The difference in cardiotoxicity and myelotoxicity between the two treatment arms and consequently the longer duration of treatment probably accounts for the better survival seen in patients receiving epirubicin. However, this significant difference may represent a false-positive observation due to the limited number of patients studied.

In conclusion, our data indicate that in patients with advanced prostatic cancer who have relapsed after hormonal therapy a weekly epirubicin regimen may give rapid palliative results, similar to that of doxorubicin, but with fewer side effects.

We thank Mrs L. Bianciardi, librarian of the Medical Library, for preparing the references, Mr F. Parlanti, system-manager at the Electronic Center, for statistical analysis and Mr A. Torricelli, laboratory technician at the Medical Pathology Institute, for skillful assistance.

This work was supported in part by grant awarded by the Italian National Research Council (CNR) – Clinical Applications of Oncologic Research (ACRO) N. 92.02170.PF/39.

### References

AHMANN, F.R. & SCHIFMAN, R.B. (1987). Prospective comparison between serum monoclonal prostate specific antigen and acid phosphatase measurements in metastatic prostatic cancer. *J. Urol.*, **137**, 431–434.

BERRY, W.R., LASZLO, J., COX, E., WALKER, A. & PAULSON, D. (1979). Prognostic factors in metastatic and hormonally unresponsive cancer of the prostate. *Cancer*, **44**, 763–775.

BERRY, O.A., LOWRY, O.H. & BROCK, M.J. (1946). A method for the rapid determination of alkaline phosphatase with five cubic millimeters of serum. *J. Biol. Chem.*, **164**, 321–329.

BRAWN, P.N. & SPEIGHTS, V.O. (1989). The dedifferentiation of metastatic prostate carcinoma. *Br. J. Cancer*, **59**, 85–88.

CANIGGIA, A. & VATTIMO, A. (1980). Kinetics of 99m Technetium-Tin-methylene-diphosphonate in normal subjects and pathological conditions: a simple index of bone metabolism. *Calcif. Tissue Int.*, **30**, 5–13.

COLEMAN, R.E. (1991). Assessment of response to treatment. In *Bone Metastases: Diagnosis and Treatment*. Rubens, R.D. & Fogelman, I. (eds). pp. 99–120. Springer-Verlag, London.

DEWYS, W.D., BAUER, M., COLSKY, J., COOPER, R.A., COLLCH, R. & CARBONE, P.P. (1977). Comparative trial of adriamycin and 5-Fluorouracil (NSC-01893) in advanced prostate cancer. Progress Report. *Cancer Treat. Rep.*, **61**, 325–328.

EISENBERGER, M.A. & ABRAMS, J.S. (1988). Chemotherapy for prostatic carcinoma. *Semin. Urol.*, **6**, 303–310.

FAUGERE, M.C. & MALLUCHE, H.H. (1983). Comparison of different bone-biopsy techniques for qualitative and quantitative diagnosis of metabolic bone disease. *J. Bone Joint Surg.*, **65**, 1314–1318.

FOSSA, S.D., HOSBACK, G. & PAUS, E. (1990). Flutamide in hormone resistant prostatic cancer. *J. Urol.*, **144**, 1411–1414.

FRANCINI, G., BIGAZZI, S., LEONE, V. & GENNARI, C. (1988). Serum osteocalcin concentration in patients with prostatic cancer. *Am. J. Clin. Oncol.*, **11**, Suppl. 2, 83–87.

FRANCINI, G., LEONE, V., PETRIOLI, R., PAFFETTI, P., PIAZZINI, M. & GIANNÌ, G. (1989). Weekly therapy with epirubicin in advanced prostatic cancer. In *5th European Conference on Clinical Oncology*, London, 3–7 September 1989. Books of abstracts. Abs 0-0784.

FRANCINI, G., GONNELLI, S., PETRIOLI, R., CONTI, F., PAFFETTI, P. & GENNARI, C. (1992). Treatment of bone metastases with dichloromethylene bisphosphonate. *J. Clin. Oncol.*, **10**, 541–549.

FROST, H.M. (1969). Tetracycline-based histological analysis of bone remodeling. *Calcif. Tissue Res.*, **3**, 211–237.

HASSAN, S. & TURNER, P. (1983). Systolic time intervals: a review of the method in the non-invasive investigation of cardiac function in health disease and clinical pharmacology. *Post. Med. J.*, **59**, 423–434.

HETHERINGTON, J.W., SIDDALL, J.K. & COOPER, E.H. (1988). Contribution of bone scintigraphy, prostatic acid phosphatase and prostate-specific antigen to the monitoring of prostatic cancer. *Eur. Urol.*, **14**, 1–5.

HUSSAIN, M., KISH, J.A., ENSLEY, J.F., TILCHEN, E. & AL-SARRAF, M. (1991). Evaluation of 5-fluorouracil infusion (5-FUI) and cisplatin-based combination chemotherapy in the treatment of patients (pts) with D2 hormone refractory adenocarcinoma of the prostate (HRCP). *Proc. Annu. Meet. Am. Sco. Clin. Oncol.*, **10**, abs.568.

IMMERGUT, M., NORDSCHOW, C.D., TAMMES, A.R. & FLOCKS, R.H. (1966). Urinary hydroxyproline excretion in patients with metastatic carcinoma of the prostate. *J. Urol.*, **96**, 570–572.

JACOBS, S.C., PIKNA, D. & LAWSON, R.K. (1979). Prostatic osteoblastic factor. *Invest. Urol.*, **17**, 195–198.

KAPLAN, H.S.V. & MEIER, P. (1958). Non parametric estimation from incomplete observation. *Am. Stat. Assoc. J.*, **53**, 457–481.

KLEIN, L.A. (1979). Prostatic carcinoma. *N. Engl. J. Med.*, **300**, 824–833.

LEVENSON, R.M., SAUERBRUNN, B.J.L., BATES, H.R., NEWMAN, R.D., EDDY, J.L. & IHDE, D.C. (1983). Comparative value of bone scintigraphy and radiography in monitoring tumor response in systemically treated prostatic carcinoma. *Radiology*, **146**, 513–518.

MOON, T.D. & SLOAME, B.B. (1989). Prostatic adenocarcinoma. Carcinogenesis and growth. *J. Am. Geriatr. Soc.*, **37**, 55–64.

NERI, B., NERI, G.C. & BANDINELLI, M. (1988). Differences between carnitine derivatives and coenzyme Q10 in preventing *in vitro* doxorubicin-related cardiac damages. *Oncology*, **45**, 242–246.

NEWLING, D.W.W. (1985). Criteria for response to treatment of metastatic prostatic cancer. *Prog. Clin. Biol. Res.*, **185A**, 205–220.

PETO, R., PIKE, M.C., ARMITAGE, P., BRESLOW, N.E., COX, D.R., HOWARD, S.V., MANTEL, N., MCPHERSON, K., PETO, J. & SMITH, P.G. (1977). Design and analysis of randomized clinical trials requiring prolonged observations of each patient. II. Analysis and examples. *Br. J. Cancer*, **35**, 1–39.

PRICE, P.A., WILLIAMSON, M.K. & LOTHRINGER, J.W. (1981). Origin of the vitamin K-dependent bone protein found in plasma and its clearance by kidney and bone. *J. Biol. Chem.*, **256**, 12760–12766.

PROCKOP, D.J. & UDENFRIEND, S. (1960). A specific method for the analysis of hydroxyproline in tissues and urine. *Anal. Biochem.*, **1**, 228–239.

REIF, A.R., SCHLESINGER, R.M., FISH, C.A. & ROBINSON, C.M. (1973). Acid phosphatase isoenzymes in cancer of the prostate. *Cancer*, **31**, 689–699.

SCHER, H.I., CURLEY, T., GELLER, N., ENGSTROM, C., DERSHAW, D.D., LIN, S.Y. & FITZPATRICK, K. (1990). Trimetrexate in prostatic cancer: preliminary observations on the use of prostate-specific antigen and acid phosphatase as a marker in measurable hormone-refractory disease. *J. Clin. Oncol.*, **8**, 1830–1838.

SCOTT, J. & HUSKISSON, E.C. (1976). Graphic representation of pain. *Pain*, **2**, 175–184.

SHARIFI, R. & SOLOWAY, M. (1990). Clinical study of leuprolide depot formulation in the treatment of advanced prostate cancer. The Leuprolide Study Group. *J. Urol.*, **143**, 68–71.

SLACK, M.H. & MURPHY, G.P. (1984). Criteria for evaluating patient responses to treatment modalities for prostatic cancer. *Urol. Clin. North Am.*, **11**, 337–342.

TORTI, F.M., ASTON, D., LUM, B.L., KOHLER, M., WILLIAMS, R., SPAULDING, J.T., SHORTLIFFE, L. & FREIHA, F.S. (1983). Weekly doxorubicin in endocrine-refractory carcinoma of the prostate. *J. Clin. Oncol.*, **1**, 477–482.

TORTI, F.M., BRISTOW, M.M., LUM, B.L., CARTER, S.K., HOWES, F.J., MITCHELL, E.P. & BILLINGHAM, M.E. (1986). Cardiotoxicity of epirubicin and doxorubicin: assessment by endomyocardial biopsy. *Cancer Res.*, **46**, 3722–3727.

VON-HOFF, D.D., ROZENCWEIG, M. & PICCART, M. (1982). The cardiotoxicity of anticancer agents. *Semin. Oncol.*, **1**, 23–33.

WAJSMAN, Z., CHU, T.M., BROSS, D., SAROFF, J., MURPHY, G.P., JOHNSON, D.E., SCOTT, W.W., GIBBONS, R.P., PROUT, G.R. & SCHMIDT, J.D. (1978). Clinical significance of serum alkaline phosphatase isoenzyme levels in advanced prostatic carcinoma. *J. Urol.*, **119**, 244–250.

WEIBEL, E.R. (1979). Sterological methods. I. Practical methods for biological morphometry. Academic Press: London.

WORLD HEALTH ORGANIZATION (1979). *WHO Handbook for Reporting Results of Cancer Treatment*. World Health Organization: Geneve.